# Posterior Circulation Ischemic Stroke Occurring After ChAdOx1 nCoV-19/AZD1222 Vaccination Without Evidence of Vaccine-Induced Immune Thrombotic Thrombocytopenia: A Case Report and Focused Narrative Review

**DOI:** 10.3390/jcm15145487

**Published:** 2026-07-13

**Authors:** Félix Bermejo-Pareja, Cristina Ramo-Tello, Julián Benito-León

**Affiliations:** 1 Group of Neurodegenerative Diseases, Hospital Universitario 12 de Octubre Research Institute (Imas12), 28041 Madrid, Spain; fbp.gijon@yahoo.es; 2 Department of Neurology, 12 de Octubre University Hospital, 28041 Madrid, Spain; cristina.ramo@salud.madrid.org; 3 Department of Medicine, Faculty of Medicine, Complutense University of Madrid, 28040 Madrid, Spain; 4 Network Center for Biomedical Research in Neurodegenerative Diseases (CIBERNED), 28031 Madrid, Spain

**Keywords:** ischemic stroke, posterior circulation, cerebellar infarction, ChAdOx1 nCoV-19, AZD1222, COVID-19 vaccination, temporal association, TOAST classification, VITT, pharmacovigilance, narrative review

## Abstract

**Background:** Ischemic stroke has been reported rarely after coronavirus disease 2019 (COVID-19) vaccination, most clearly in association with vaccine-induced immune thrombotic thrombocytopenia (VITT). Nevertheless, temporal proximity alone cannot establish causality, particularly when conventional vascular risk factors are present. We report a case of posterior circulation ischemic stroke occurring 36–48 h after first-dose ChAdOx1 nCoV-19/AZD1222 vaccination without VITT and provide a focused narrative review of the clinical, epidemiological, and mechanistic evidence regarding post-vaccination stroke. **Methods:** Clinical, laboratory, and neuroimaging data were reviewed retrospectively. A focused narrative literature review was performed to contextualize VITT-related and non-VITT ischemic stroke after COVID-19 vaccination, pharmacovigilance signals, population-based evidence, and proposed biological mechanisms. **Results:** A 63-year-old man with well-controlled hypertension and a remote history of smoking developed systemic symptoms within 24 h of first-dose ChAdOx1 nCoV-19/AZD1222 vaccination, followed by severe hypertension and acute posterior circulation neurological symptoms 36–48 h after vaccination. Neuroimaging showed an acute left cerebellar infarct extending into the middle cerebellar peduncle, with approximately 50% stenosis of the intradural left vertebral artery. Platelet counts remained normal, D-dimer and anti-PF4 antibody testing were normal, SARS-CoV-2 polymerase chain reaction (PCR) testing was negative, and routine cardiac evaluation did not identify a definite cardioembolic source. **Conclusions:** This case highlights an early posterior circulation ischemic stroke temporally associated with ChAdOx1 nCoV-19/AZD1222 vaccination in the absence of VITT. Although individual causality cannot be established from a single case, careful reporting of such presentations may improve clinical recognition, etiological evaluation, and pharmacovigilance.

## 1. Introduction

Coronavirus disease 2019 (COVID-19) began as an outbreak of pneumonia of presumed viral origin in Wuhan, Hubei, China, in December 2019, likely originating from a bat-borne virus reservoir [1,2]. The disease was initially characterized by pulmonary symptoms, fever, cough, and myalgia, and was later attributed to SARS-CoV-2 [3]. On 11 March 2020, the World Health Organization declared COVID-19 a pandemic [2,4]. Severe acute respiratory syndrome coronavirus 2 (SARS-CoV-2) is an RNA virus of the betacoronavirus family. Its genome encodes four main structural proteins: the spike surface glycoprotein, membrane protein, envelope protein, and nucleocapsid protein. The receptor-binding domain of the spike protein binds to angiotensin-converting enzyme 2 (ACE2) on the plasma membrane, initiating receptor-mediated endocytosis and viral entry [3,4].

The rapid spread of the virus and the lack of curative drugs prompted the urgent development of vaccines [4,5,6,7,8]. By August 2020, many vaccine platforms and candidate drugs were under investigation [3,4]. COVID-19 vaccine development was scientifically rapid and included attenuated or inactivated-virus vaccines, viral-vector vaccines such as ChAdOx1 nCoV-19/AZD1222 (COVID-19 Vaccine AstraZeneca; later Vaxzevria; hereafter ChAdOx1/AZD1222), and Ad26.COV2.S, Sputnik, and mRNA vaccines such as mRNA-1273 and BNT162b2 [4,5,6,7,8,9].

The ChAdOx1/AZD1222 vaccine demonstrated efficacy in phase III clinical trials [9], as did other marketed vaccines [10,11,12]. However, very rare adverse events may become apparent only after large-scale population rollout; therefore, real-world adverse events following immunization (AEFIs) were primarily characterized in phase IV population-based studies and pharmacovigilance systems. For viral-vector vaccines, a newly described syndrome termed vaccine-induced immune thrombotic thrombocytopenia (VITT), also referred to as thrombosis with thrombocytopenia syndrome (TTS), was identified [13,14]. VITT may present with venous thrombosis, secondary hemorrhage, and arterial thrombosis, including myocardial infarction and stroke [13,14]. The mechanism is incompletely understood but involves platelet factor 4 antibodies [15].

These unexpected events led to temporary regulatory actions in Europe [16]; European regulators later emphasized their rarity and the positive benefit-risk balance [17], although ChAdOx1/AZD1222 was not authorized in the United States [18]. Subsequent pharmacovigilance studies, particularly the European study by Cari et al. [19], showed that stroke as an AEFI is very infrequent after COVID-19 vaccination, although more frequent than after influenza vaccination in some analyses [20]. A large Mexican AEFI series reported that most strokes occurred after viral-vector vaccines, especially ChAdOx1/AZD1222, and after the first dose, but events were also reported with other vaccines [21].

In summary, ischemic stroke occurring after COVID-19 vaccination is rare at the population level and is often reported in patients with conventional vascular risk factors. This article presents a case of posterior circulation ischemic stroke occurring shortly after ChAdOx1/AZD1222 vaccination, without evidence of VITT, and reviews the literature, explicitly separating temporal association, biological plausibility, hypothesis generation, and causal inference.

## 2. Methods: Case Report and Focused Narrative Review

This article combines a single case report with a focused narrative review. The case report component was prepared in accordance with the CARE reporting guideline; the completed CARE checklist is provided in Supplementary File S1.

No commercial laboratory reagents, research-specific devices, cell lines, or prospectively collected samples were used in this retrospective case report. Figures 2–4 were prepared by the authors using FigureLabs (https://www.figurelabs.ai/; accessed on 16 May 2026), a web-based image-creation platform.

MEDLINE/PubMed and Google Scholar were searched and re-checked during revision through 30 May 2026. Search concepts were combined using Boolean operators and included: (COVID-19 OR SARS-CoV-2) AND (ischemic stroke OR arterial stroke OR cerebrovascular disease); (COVID-19 vaccine OR ChAdOx1 OR AZD1222 OR Vaxzevria OR adenoviral-vector vaccine) AND (stroke OR arterial thrombosis OR cerebral venous sinus thrombosis OR VITT OR thrombosis with thrombocytopenia syndrome); and (post-COVID condition OR post-COVID syndrome OR post-vaccination syndrome OR post-acute COVID-19 vaccination syndrome) AND (stroke OR endotheliopathy OR microvascular injury).

Eligibility and selection: Priority was given to systematic reviews, meta-analyses, nationwide or multinational cohort studies, self-controlled case series/case-crossover studies, active surveillance studies, clinically informative case reports, and authoritative guidance on pharmacovigilance or causality assessment. Exclusion criteria were duplicate reports, non-peer-reviewed commentary without primary data or clinically useful synthesis, articles with insufficient clinical detail for the question addressed, and studies unrelated to ischemic stroke, VITT/TTS, post-vaccination cerebrovascular events, or relevant mechanisms. Titles and abstracts were screened first, followed by full-text review for potentially relevant items. The final reference list represents the included sources.

## 3. Case Presentation and Review Findings

### 3.1. Case Illustration

A 63-year-old man with a history of well-controlled arterial hypertension for approximately nine years, Gilbert syndrome, and remote tobacco use (cessation approximately 12 years earlier) received the first dose of the adenoviral-vector COVID-19 vaccine ChAdOx1 nCoV-19/AZD1222 (COVID-19 Vaccine AstraZeneca; later Vaxzevria). There was no known previous thrombosis, autoimmune disease, or recent heparin exposure. Relevant family history included cerebrovascular and cardiovascular disease.

Within the first 24 h after vaccination, the patient developed fever, headache, malaise, marked fatigue, and nocturnal chills. Approximately 36–48 h after vaccination, abrupt severe hypertension, with a reported peak blood pressure of approximately 220/115 mmHg, was followed by nausea and vomiting and prominent vestibulocerebellar symptoms, including vertigo, gait instability, dysarthria, and diplopia. These symptoms prompted emergency evaluation.

Initial neurological examination showed horizontal gaze-evoked nystagmus, worse on left gaze, with a vertical component most evident on upgaze; impaired abduction of the left eye with binocular horizontal diplopia; left pseudo-peripheral facial weakness; abolished gag reflex on stimulation of the left pharyngeal arch; left-sided limb dysmetria; mild dysarthria; and gait ataxia. The National Institutes of Health Stroke Scale score at presentation was 5.

Non-contrast head computed tomography demonstrated a basal corticosubcortical hypodensity in the left cerebellar hemisphere compatible with acute ischemia in the posterior inferior cerebellar artery territory, together with chronic lacunar infarcts in the bilateral caudate nuclei, right thalamus, and left corona radiata, and mild periventricular leukoaraiosis. Computed tomography angiography of the head and neck demonstrated approximately 50% stenosis of the intradural segment of the left vertebral artery, without large-vessel occlusion. The dural venous sinuses were patent.

**Figure 1:**
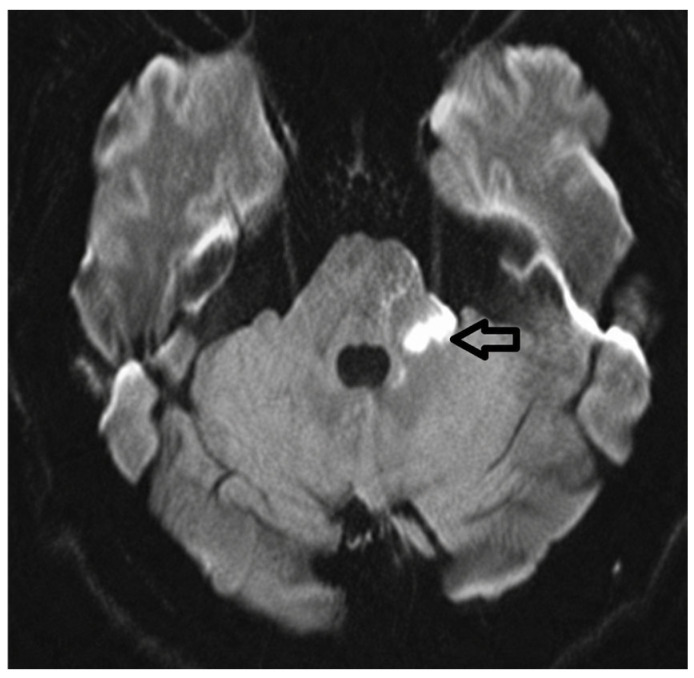
Case illustration. Axial diffusion-weighted MRI image showing a left cerebellar infarct as hyperintensity (arrow) extending into the middle cerebellar peduncle.

Brain magnetic resonance imaging (MRI) confirmed acute ischemic lesions in the left cerebellar hemisphere extending into the middle cerebellar peduncle (Figure 1).

The patient underwent an extensive diagnostic evaluation. Chest radiography, 12-lead electrocardiography, inpatient cardiac monitoring, transthoracic echocardiography, head and neck vascular imaging, routine biochemistry, complete blood count, coagulation testing, SARS-CoV-2 PCR, and VITT/TTS-focused testing did not identify a definite cardioembolic, infectious, venous-thrombotic, or immune-thrombotic cause. Thrombolysis and thrombectomy were not indicated, and he was treated with antihypertensive therapy and dual antiplatelet therapy. After 12 days on the neurology ward, he was transferred to inpatient rehabilitation with partial neurological improvement. Platelet counts remained within the reference range; anti-PF4 antibody testing and quantitative D-dimer levels were normal; and the clinical picture did not support VITT.

During the following months, the patient reported persistent cognitive and affective symptoms, including impaired concentration, recent-memory difficulties, marked mental fatigue, sleep disturbance, and depressive symptoms, with substantial occupational impairment. These symptoms were compatible with a brain-fog-like post-stroke neurocognitive syndrome. At 5-year follow-up, the focal neurological deficit and post-stroke depression had largely improved, but memory difficulties, apathy/fatigue, and gait disturbance persisted.

The case timeline is summarized in Table 1.

### 3.2. Etiological Evaluation and Competing Stroke Mechanisms

The patient underwent a broad acute and follow-up etiological evaluation. Prolonged cardiac rhythm monitoring, transesophageal echocardiography, comprehensive extracranial and intracranial vascular imaging, thrombophilia testing, inflammatory biomarker assessment, lipid and glycemic profiling, SARS-CoV-2 testing, and VITT/TTS evaluation did not identify a definite cardioembolic, inflammatory, thrombophilic, metabolic, infectious, venous-thrombotic, or VITT/TTS-related mechanism. The relevant vascular background findings were severe hypertension at presentation, chronic lacunar infarcts/mild leukoaraiosis, and approximately 50% stenosis of the intradural left vertebral artery, which could represent a potential but not definitive artery-to-artery mechanism. The close temporal relationship with first-dose ChAdOx1 nCoV-19/AZD1222 vaccination, together with systemic post-vaccination symptoms and abrupt severe hypertension, makes a temporally associated post-vaccination vascular trigger clinically plausible, but not proven. Therefore, using the TOAST framework [22], the most appropriate classification is ischemic stroke of undetermined etiology after comprehensive evaluation, while acknowledging the vaccination-related temporal association and the vascular background findings as relevant interpretive considerations (Table 2).

### 3.3. Case Commentary: Temporal Association, Etiological Uncertainty, and Causality

This case should be described as a posterior circulation ischemic stroke occurring after ChAdOx1/AZD1222 vaccination. The temporal relationship is clinically notable because systemic symptoms preceded the neurological event, SARS-CoV-2 PCR was negative, VITT/TTS was not supported, and extensive etiological evaluation did not identify a definite cardioembolic, inflammatory, thrombophilic, metabolic, infectious, venous-thrombotic, or VITT/TTS-related cause. The patient also had vascular background findings, including hypertension, vertebral-artery stenosis, chronic lacunar infarcts, and leukoaraiosis. These findings do not establish a definitive alternative etiology, but they require caution in attributing individual causality to vaccination. Therefore, the case is hypothesis-generating and should not be presented as proof of vaccine-induced stroke.

The temporal sequence does not allow determination of whether severe hypertension acted as a trigger for the posterior circulation stroke, represented an early physiological response to evolving ischemia, reflected pain/stress/autonomic activation, or interacted with pre-existing vertebral-artery disease. This uncertainty is central to the interpretation of the case.

Previously reported acute arterial ischemic strokes occurring after COVID-19 vaccination include cases with VITT after ChAdOx1/AZD1222 or Ad26.COV2.S vaccination and cases without VITT after several vaccine platforms [23,24,25,26,27,28,29,30,31,32,33,34]. Table 3 summarizes illustrative reports together with the present case. The table is descriptive and should not be read as evidence that each reported stroke was causally related to vaccination.

### 3.4. Causality Assessment, Pharmacovigilance, and Epidemiological Context

Assessment of a suspected adverse event following immunization requires explicit separation of temporal association, biological plausibility, and causal inference. Black et al. [35], on behalf of the Brighton Collaboration, emphasized that even large phase III vaccine trials have limited power to detect very rare adverse events and that post-introduction vaccine-safety assessment should integrate standardized case evaluation, background rates, and active surveillance. For an individual case, temporal proximity is necessary but not sufficient; recurrence on rechallenge, a distinctive syndrome, specific laboratory evidence, or consistent epidemiological excess would strengthen causal inference, none of which is available here.

In the present case, no specific laboratory marker establishes vaccine causality, and no vaccine rechallenge occurred. The most defensible interpretation is that the stroke occurred in close temporal association with vaccination and should be considered a pharmacovigilance signal or hypothesis-generating observation. The clinical chronology, absence of SARS-CoV-2 infection, absence of VITT/TTS, and lack of a definite cardioembolic source support the reporting and discussion; the patient’s vascular risk factors, vertebral artery stenosis, and chronic small-vessel disease prevent attribution of individual causality.

At the population level, the most informative data come from active surveillance, linked electronic health record cohorts, self-controlled case series analyses, and case-crossover studies rather than spontaneous reporting systems. Such designs better address background rates, time-varying infection risk, confounding by indication, and healthy-vaccinee bias [35,36,37,38,39,40,41,42,43,44,45,46,47,48,49].

Passive pharmacovigilance systems remain essential for early signal detection, but they should not be interpreted as incidence studies or proof of causality. The Vaccine Adverse Event Reporting System (VAERS) explicitly states that reports alone cannot determine whether a vaccine caused or contributed to an adverse event and that the number of reports alone cannot be interpreted as evidence of causality, frequency, or rates [50]. EudraVigilance similarly emphasizes that suspected side-effect reports describe medical events observed after medicine use, not necessarily events caused by the medicine, and that robust conclusions require scientific assessment of all available data [51].

Large observational studies provide a more balanced context. In the 46-million-adult England cohort, overall rates of major arterial and venous events were lower after vaccination with either ChAdOx1-S or BNT162b2 after adjustment. In contrast, small absolute excess risks of intracranial venous thrombosis and thrombocytopenia were observed after ChAdOx1-S in adults younger than 70 years [49]. Other self-controlled studies and reviews report neutral, conflicting, or platform-specific findings and generally indicate that thrombotic and neurological risks after SARS-CoV-2 infection exceed those observed after vaccination [39,40,41,42,43,44,45,46,47].

The survey by Torabi et al. [44], which included more than two million individuals in Wales who received ChAdOx1/AZD1222 or BNT162b2, is discussed as one example of a self-controlled design. Its findings should be interpreted with other nationwide and multinational analyses rather than as standalone proof of causality. McKeigue et al. [45], using a case-crossover methodology and neuroimaging ascertainment in Scotland, likewise illustrate the value of designs that reduce between-person confounding when evaluating rare post-vaccination thrombotic events.

Analyses comparing adverse-event profiles across vaccine types can generate useful hypotheses but are sensitive to reporting bias and differential vaccine deployment. The EudraVigilance analysis by Cari et al. [19] suggested higher reporting rates of several cardiovascular, neurological, and thrombotic events following viral vector vaccines than following mRNA vaccines. These findings are best viewed as signal-detection data requiring confirmation through active surveillance and population-based designs, rather than as direct estimates of comparative incidence or individual causality [50,51].

Reported stroke-event rates vary substantially across datasets and study designs. In EudraVigilance, reporting rates for stroke were higher for ChAdOx1/AZD1222 and Ad26.COV2.S than for BNT162b2 in the analysis by Cari et al. [19], whereas the Mexican nationwide descriptive study estimated stroke after vaccination as a very rare event (0.71 cases per million administered doses) [21]. These differences likely reflect differences in case ascertainment, background risk, vaccine allocation, reporting behavior, and denominator definitions.

Thus, the epidemiological evidence is mixed but consistently supports two points relevant to this case: first, ischemic stroke shortly after vaccination is very uncommon; second, case reports alone cannot distinguish coincidence from causality when conventional vascular risk factors and plausible competing mechanisms are present [21,39,40,41,42,43,44,45,46,47,48,49,52,53,54,55].

Pathological and mechanistic studies may support biological plausibility in selected settings, but they cannot, by themselves, establish causation in this patient. Pathology-proven cases and short autopsy or biopsy series after vaccination have been reported [56,57,58,59,60,61,62,63,64,65,66,67], while negative necropsy series are also part of the evidence base [65,66]. These observations should be interpreted cautiously and in conjunction with population-level evidence.

Post-COVID-19 syndrome (PCS), Neuro-COVID, and post-COVID-19 vaccination syndrome (PCVS) are distinct entities with different evidentiary foundations. PCS after SARS-CoV-2 infection has consensus definitions and substantial epidemiological literature, whereas PCVS is not yet a universally accepted clinical diagnosis and lacks standardized diagnostic criteria. Persistent fatigue, cognitive symptoms, depression, sleep disturbance, and gait impairment in the present patient may be explained by post-stroke sequelae and should not be attributed to PCVS without additional evidence [68,69,70,71,72,73,74,75,76,77,78,79,80,81,82,83,84,85,86,87,88,89,90,91,92,93,94,95].

Mechanistic discussion should therefore be evidence-graded. VITT/TTS is a well-established immune-mediated thrombotic syndrome associated with adenoviral vector vaccines and anti-PF4 antibodies. By contrast, spike-protein persistence, endothelial dysfunction after vaccination, microvascular injury, immune dysregulation, and PCVS-related mechanisms remain proposed or speculative pathways in arterial ischemic stroke after vaccination without VITT. Mechanisms demonstrated in severe acute SARS-CoV-2 infection should not be directly extrapolated to vaccination without specific supporting evidence.

Taken together, the presented case should be interpreted as a hypothesis-generating observation of posterior circulation ischemic stroke occurring shortly after ChAdOx1/AZD1222 vaccination. The case supports careful pharmacovigilance reporting and illustrates diagnostic challenges, but it does not prove a vaccine-induced event. The most balanced interpretation is TOAST-classified ischemic stroke of undetermined etiology after comprehensive evaluation, with no evidence of SARS-CoV-2 infection, no evidence of VITT/TTS, no definite alternative etiology identified, and a close temporal relationship with vaccination.

## 4. Discussion

### 4.1. Stroke in Acute SARS-CoV-2 Infection

Stroke is a leading cause of disease burden worldwide. The 21st-century definition of stroke is an episode of neurological dysfunction caused by focal cerebral, spinal, or retinal infarction [96]. Stroke epidemiology varies substantially by country, sex, race, and age, with higher prevalence in less developed countries [97]. In Western countries, ischemic stroke accounts for the majority (80–90%) of cases and hemorrhagic stroke for 10–15% [98,99,100], whereas hemorrhagic stroke is more frequent in some Eastern populations, including China [101]. In broad terms, the prevalence of stroke in the general population is 1–4.9% in people older than 20 years [98]. The estimated global lifetime risk of stroke from age 25 onward is high (approximately 25%) and varies according to cardiovascular risk factors, sex, and age [99,102].

COVID-19 has been associated with increased stroke risk compared with some other viral infections, including influenza, after adjustment for relevant variables [103]. The incidence estimates vary [104]. A systematic review reported rates of around 1.4% among infected persons [105], whereas some hospital-based reviews reported rates approaching 5% [104,106]. Other studies reported lower rates [107]. The literature includes conflicting short reports [108,109,110,111,112,113,114,115,116,117,118,119,120], systematic reviews with different aims [121,122,123,124,125,126,127,128,129,130,131,132,133,134,135,136,137,138,139,140,141,142,143], and an umbrella review [144].

Clinically, a large collaborative investigation of acute ischemic stroke in COVID-19 reported that affected patients were younger, had more severe strokes, worse disability and mortality, more frequent large-artery involvement, and a high proportion of vertebrobasilar events compared with the general stroke population [145]. The literature also describes heterogeneous cerebrovascular phenotypes, including endotheliopathy-associated microvascular disease [146,147], small-vessel disease [138], and complex clinicopathological findings [148]. Pathological and histopathological data have also documented brain vascular lesions in selected COVID-19 patients [109,148].

Most systematic reviews, meta-analyses [121,122,123,124,125,126,127,128,129,130,131,132,133,134,135,136,137,138,139,140,141,142,143], and the umbrella review [144] indicate that stroke risk was increased among patients with COVID-19, especially severe COVID-19, with high associated mortality. Ischemic strokes, cryptogenic mechanisms, and male sex were frequent features. Children and young people also experienced increased ischemic stroke in some studies [113,114,136]. During the pandemic, hospital admissions for mild stroke and transient ischemic attack decreased in some settings, while severe presentations increased [139,141]. A recent self-controlled case series also documented an increased risk of stroke or myocardial infarction following hospitalization for community-acquired pneumonia, underscoring that post-infectious vascular risk is not specific to COVID-19 [149]. These real-world data differ from the low absolute incidence reported in clinical trials [150].

In summary, COVID-19 is associated with increased stroke risk, mainly ischemic stroke, often with cryptogenic etiology and unfavorable prognosis.

**Figure 2:**
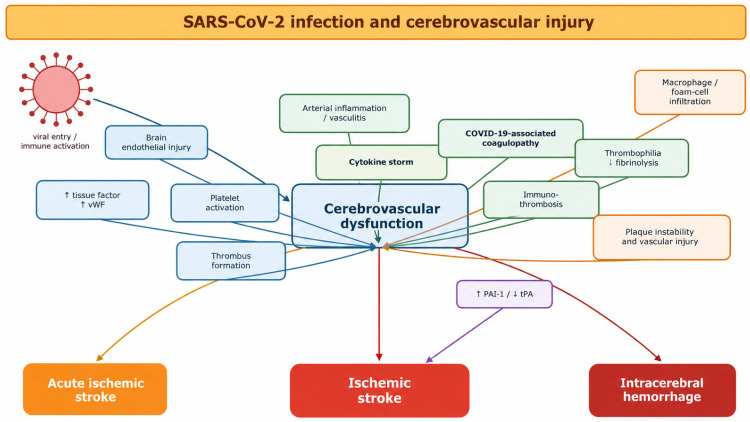
Pathophysiological mechanisms linking SARS-CoV-2 infection to ischemic and hemorrhagic stroke. SARS-CoV-2 infection may promote cerebrovascular injury through multiple, partly overlapping mechanisms, including brain endothelial damage, increased tissue factor expression, elevated von Willebrand factor levels, platelet activation, thrombus formation, arterial inflammation or vasculitis, cytokine storm, COVID-19-associated coagulopathy, immunothrombosis, a prothrombotic state with impaired fibrinolysis, and macrophage/foam-cell infiltration contributing to vascular injury and plaque instability [158,176]. These pathways may converge to increase the risk of acute ischemic stroke and intracerebral hemorrhage in acute infection; they should be distinguished from proposed mechanisms after vaccination. Abbreviations: PAI-1, plasminogen activator inhibitor-1; TF, tissue factor; tPA, tissue plasminogen activator; vWF, von Willebrand factor. Arrows indicate proposed directional relationships and convergence toward cerebrovascular dysfunction and clinical outcomes. Created by the authors using FigureLabs image-creation software (FigureLabs; https://www.figurelabs.ai/; accessed on 16 May 2026).

The causal association between SARS-CoV-2 infection and stroke is incompletely understood. Proposed mechanisms include systemic inflammation [129,151,152,153], immune-inflammatory mechanisms [69,153,154,155], a procoagulant state [151,156,157,158], thrombotic microangiopathy [138,146,147], viral persistence and immune-induced derangements [159,160,161,162], spike protein-related mechanisms [151,152,163,164,165], and endothelial injury [69,163,166,167,168,169,170,171,172,173,174,175]. A focused review of the hematological manifestations of COVID-19 summarizes these, including D-dimer elevation, activation of coagulation/fibrinolysis, endothelial dysfunction, increased von Willebrand factor/factor VIII activity, and hypercoagulability in severe disease [158]. These mechanisms are relevant to SARS-CoV-2 infection but should not be automatically extrapolated to vaccination without direct clinical evidence. These mechanisms are summarized in Figure 2.

### 4.2. Stroke After the Acute Phase: PCS and Neuro-COVID

A review of stroke after the acute phase of SARS-CoV-2 infection requires consideration of a long time frame, ranging from four weeks to several years. Several terms have been used, including post-acute COVID-19, post-COVID-19 condition, and post-COVID-19 syndrome (PCS) [68,69,70,71,72,177,178]. Definitions have also been proposed by major organizations and Delphi panels [179,180]. Because terminology and content remain heterogeneous, this review uses PCS descriptively to refer to the period following the acute infection, generally after 4 weeks.

PCS symptoms and signs can be diverse. Many definitions require that symptoms last approximately 3 months and begin after the acute infection [68,69]. Presentations may be predominantly neurological, including brain fog [181,182], or may involve gastrointestinal, respiratory, musculoskeletal, or multisystem symptoms [68,179]. Fatigue is a common accompanying symptom. PCS can affect multiple organ systems and may cause severe and prolonged impairment. It appears particularly frequent after severe SARS-CoV-2 infection and has been described as a multisystem post-viral disease rather than a group of pathologically independent subsyndromes [69,82,183,184].

**Figure 3:**
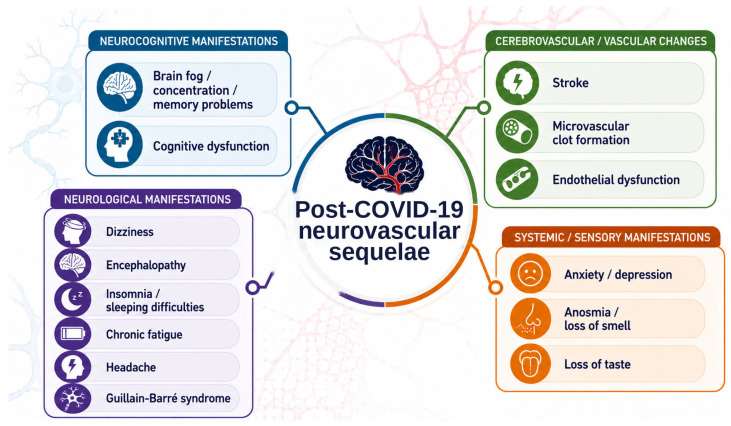
Post-COVID-19 neurovascular sequelae. Schematic representation of neurocognitive, neurological, cerebrovascular/vascular, and systemic/sensory manifestations described in post-COVID-19 syndrome and Neuro-COVID, including brain fog, cognitive dysfunction, dizziness, encephalopathy, insomnia or sleeping difficulties, chronic fatigue, headache, Guillain-Barré syndrome, stroke, microvascular clot formation, endothelial dysfunction, anxiety/depression, anosmia, and loss of taste. Created by the authors using FigureLabs image-creation software (FigureLabs; https://www.figurelabs.ai/; accessed on 16 May 2026).

The main neurological and neurovascular manifestations relevant to PCS and Neuro-COVID, including brain fog, cognitive dysfunction, microvascular clot formation, endothelial dysfunction, and stroke, are summarized in Figure 3.

To evaluate whether patients who have experienced SARS-CoV-2 infection have increased stroke risk after the acute period, two scenarios should be distinguished: stroke risk during a long follow-up period after infection and stroke risk in the context of other PCS manifestations, including general health or neurological disorders. This distinction is partly academic because many clinical series do not separate these categories.

Long-term investigations after SARS-CoV-2 infection [74,77,185,186,187,188,189,190,191], reviews [192], and systematic reviews [193] generally report an increased risk of stroke, similar to PCS surveys [74,75,77], reviews [69,73,76,194,195], and systematic reviews [81,84]. Specific topics include cardiovascular disease and stroke in PCS [80,196], SARS-CoV-2 persistence [197], microcirculatory impairment in pediatric PCS [198], and Neuro-COVID-19 [71,199,200]. Other surveys and reviews addressed broader cardiac, neurological, or general health outcomes and provided variable or limited stroke-specific data [79,201,202,203,204,205,206,207,208,209,210,211,212,213,214].

Examples of reported risk estimates include a cohort of 236,379 recovered patients in which, at six months, intracranial hemorrhage occurred in 0.56% and ischemic stroke in 2.10%; among severe patients who required intensive therapy, estimates increased to 2.66% for intracranial hemorrhage and 6.92% for ischemic stroke [77]. In a review of more than 23 million survivors of COVID-19, ischemic stroke at nine months occurred in 4.40 per 1000 compared with 3.25 per 1000 controls [193]. In PCS, a systematic review reported a pooled stroke risk 1.71 times higher than in non-COVID-19 patients [81]. In more than four million participants, ischemic stroke risk remained increased beyond two years [188]. By contrast, individuals with community-managed SARS-CoV-2 infection did not show increased long-term stroke risk in one study [191].

Age, cardiovascular risk factors, and severity of SARS-CoV-2 infection appear to modify stroke risk after COVID-19 [176,191]. Mild or community-managed SARS-CoV-2 infection may be associated with a smaller or no increase in long-term stroke risk in some studies [191]. The modifying effects of vaccination status, early antiviral treatment, and genetic predisposition require further study.

Before turning to vaccination-related syndromes, it is important to distinguish Neuro-COVID from post-vaccination chronic illness with post-COVID-like features and complex chronic adverse events following immunization, which are discussed separately below [86,87].

Neuro-COVID-19 is a clinically important entity characterized primarily by cognitive sequelae, including memory loss, anxiety, brain fog, microvascular effects, and fatigue [70,71,88,89,90,202,204,205]. Some authors consider it a manifestation of PCS with brain vascular pathology and immune-mediating molecules contributing to its pathogenesis [90]. Reliable data on stroke incidence specifically within Neuro-COVID-19 are lacking.

### 4.3. Vaccination, PCVS, and Stroke Risk

The epidemiological data reviewed above indicate that ischemic stroke occurring within 30 days of COVID-19 vaccination is very uncommon across vaccine platforms. Available reports suggest that thrombotic events with thrombocytopenia are most strongly associated with adenoviral vector vaccines. In contrast, evidence for non-VITT arterial ischemic stroke is inconsistent and limited by confounding, background stroke incidence, and case ascertainment. Consequently, the present case should be interpreted in light of conventional stroke mechanisms and population-level data, rather than as direct evidence of vaccine causality.

PCVS [61,68,69,70,71,85,87,91,92,93,94,95,199] is not codified in formal diagnostic nosology and lacks universally accepted diagnostic criteria. In this review, PCVS is used descriptively and cautiously rather than as an established medical diagnosis. Reported symptoms are heterogeneous and may overlap with post-stroke sequelae, depression, sleep disturbance, dysautonomia, medication effects, and other conditions. Therefore, persistent cognitive symptoms, fatigue, mood symptoms, or gait disturbance in this patient are discussed primarily as possible post-stroke sequelae rather than evidence of PCVS. The principal SARS-CoV-2-related entities and their reported cerebrovascular manifestations are summarized in Table 4.

PCVS and Neuro-COVID-19 have been hypothesized to involve vascular endothelial injury or microvascular dysfunction [61,70,71,95,215,216], but no clear increase in clinical stroke manifestations has been demonstrated specifically for PCVS. This distinction is important for interpreting the present patient, whose chronic symptoms can be explained by the index posterior circulation stroke and its neuropsychiatric sequelae.

### 4.4. Shared and Distinct Pathophysiological Mechanisms

The syndromic entities described in this review have overlapping but distinct pathophysiological explanations. Severe acute SARS-CoV-2 infection is strongly associated with inflammation, coagulopathy, endothelial injury, and thrombosis. VITT/TTS after adenoviral-vector vaccination is a well-defined immune-mediated anti-PF4 syndrome. By contrast, non-VITT arterial ischemic stroke after vaccination, PCVS-related vascular injury, and spike-persistence mechanisms remain proposed pathways with variable and often indirect evidence [13,14,15,16,39,47,56,57,58,68,69,70,71,83,92,104,120,121,122,123,124,151,152,158,160,164,166,176,199,217].

**Figure 4:**
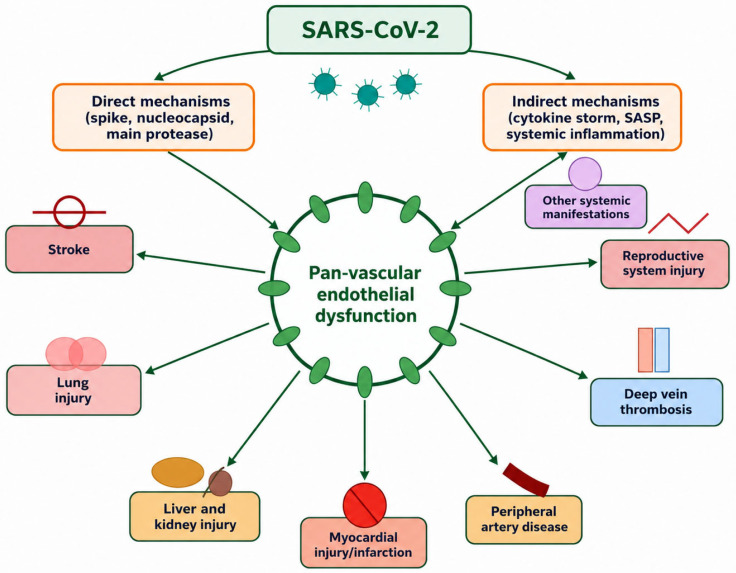
SARS-CoV-2 infection, endothelial dysfunction, and multi-organ injury. Direct SARS-CoV-2 infection mediated by viral proteins, such as the spike protein, nucleocapsid protein, and main protease, and indirect mechanisms related to systemic inflammation, cytokine storm, and the senescence-associated secretory phenotype (SASP), may induce endothelial dysfunction throughout the pan-vasculature. This endothelial injury may contribute to multi-organ involvement, including stroke, lung injury, liver and kidney injury, myocardial injury/infarction, peripheral artery disease, deep vein thrombosis, reproductive system injury, and other systemic manifestations. Abbreviation: SASP, senescence-associated secretory phenotype. Created by the authors using FigureLabs image-creation software (FigureLabs; https://www.figurelabs.ai/; accessed on 16 May 2026).

A pan-vascular model of SARS-CoV-2-related endothelial dysfunction and multi-organ injury is shown in Figure 4.

Table 5 summarizes major mechanisms and grades their relevance to the present case. The categories are necessarily artificial because inflammation, immune reaction, coagulation, and vascular or microvascular lesions may be interdependent consequences of infection or vaccination [69,71]. The table distinguishes established mechanisms (e.g., VITT/TTS and COVID-19-associated coagulopathy) from proposed biological pathways and speculative hypotheses.

**Figure 5:**
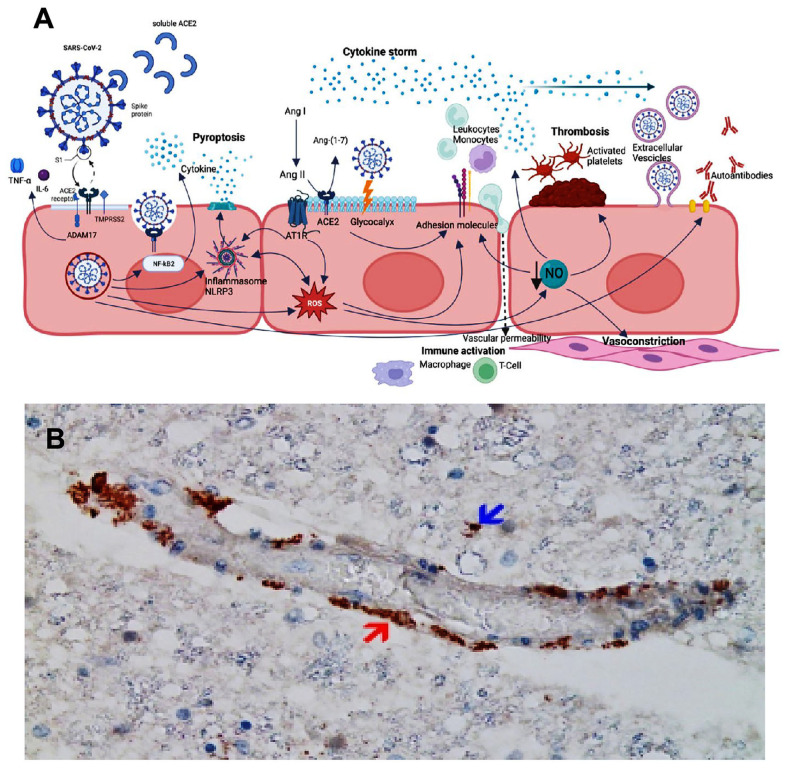
Endothelial dysfunction in SARS-CoV-2 infection and tissue spike-protein immunoreactivity in a post-vaccination autopsy case. (**A**) Schematic summary of the principal endothelial dysfunction pathways associated with SARS-CoV-2 infection, including ACE2/TMPRSS2/ADAM17-mediated viral entry and ACE2 shedding, renin–angiotensin system imbalance with Ang II/AT1R predominance, NLRP3 inflammasome activation, reactive oxygen species generation, pyroptosis, cytokine release, glycocalyx disruption, leukocyte recruitment, extracellular-vesicle signaling, reduced nitric oxide bioavailability, vascular permeability, vasoconstriction, platelet activation, and thrombosis. Adapted from Santoro et al. [241], distributed under the terms of the Creative Commons Attribution 4.0 International License. (**B**) Frontal-lobe immunohistochemical staining from the autopsy case reported by Mörz showing SARS-CoV-2 spike subunit 1 immunoreactivity as brown granular deposits in capillary endothelial cells (red arrow) and in individual glial cells (blue arrow)—original magnification: 200×. Adapted from Mörz [57], distributed under the terms of the Creative Commons Attribution 4.0 International License. Abbreviations: ACE2, angiotensin-converting enzyme 2; ADAM17, a disintegrin and metalloproteinase 17; Ang I, angiotensin I; Ang-(1–7), angiotensin-(1–7); Ang II, angiotensin II; AT1R, angiotensin II type 1 receptor; IL-6, interleukin 6; NLRP3, NOD-, LRR- and pyrin domain-containing protein 3; NO, nitric oxide; ROS, reactive oxygen species; SARS-CoV-2, severe acute respiratory syndrome coronavirus 2; TMPRSS2, transmembrane serine protease 2; TNF-α, tumor necrosis factor alpha.

After reviewing the mechanisms summarized in Table 5, the most frequently discussed mechanisms are coagulopathy, endothelial injury, immune dysregulation, microvascular dysfunction, and spike-protein-related effects. These mechanisms differ substantially in evidentiary strength. VITT/TTS and severe COVID-19-associated coagulopathy are well established, whereas non-VITT post-vaccination arterial stroke mechanisms remain incompletely defined. Careful characterization of rare adverse events is compatible with a pro-vaccination and vaccine-safety framework [240]. Figure 5 highlights endotheliopathy in relation to infection and selected mechanistic observations.

## 5. Conclusions

The presented case is best interpreted as a posterior circulation ischemic stroke occurring shortly after ChAdOx1/AZD1222 vaccination without evidence of VITT/TTS. The temporal relationship, systemic post-vaccination symptoms, severe hypertension at the onset of neurological symptoms, absence of SARS-CoV-2 infection, and extensive negative etiological evaluation justify pharmacovigilance reporting and hypothesis generation. Individual causality cannot be established from a single case, but no definite alternative etiology was identified despite broad evaluation.

Clinically, this case emphasizes that patients with acute neurological symptoms after vaccination require standard evidence-based stroke evaluation and treatment, with targeted VITT/TTS testing when timing and laboratory findings are compatible. Future studies should prioritize active surveillance, self-controlled designs, neuroradiological adjudication, standardized laboratory reporting, and mechanistic investigations that distinguish established syndromes from proposed pathways and speculative hypotheses.

## Figures and Tables

**Table 1:** Clinical timeline of the presented case.

Approximate Time from Vaccination	Clinical Events and Key Findings
0 h (Day 0)	First dose of ChAdOx1 nCoV-19/AZD1222 administered.
~24 h	Fever, malaise, headache, marked fatigue, and nocturnal chills.
Post-vaccination day 2 (approximately 36–48 h)	Severe hypertension (approximately 220/115 mmHg) followed by nausea/vomiting, vertigo, gait instability, dysarthria, and diplopia.
Emergency evaluation	NIHSS 5. CT: acute left cerebellar hypodensity; chronic lacunes and mild leukoaraiosis. CTA: ~50% intradural left vertebral artery stenosis; no large-vessel occlusion; dural venous sinuses patent. Platelets within reference range; SARS-CoV-2 PCR negative.
During hospitalization	Brain MRI confirmed acute left cerebellar ischemia extending into the middle cerebellar peduncle. Chest radiography, ECG/inpatient rhythm assessment documented in the record, and transthoracic echocardiography did not identify a definite cardioembolic source.
VITT work-up	Platelet counts remained within the reference range; anti-PF4 antibody testing and quantitative D-dimer levels were normal; no cerebral venous sinus thrombosis was identified. The clinical picture was not typical of VITT.
Discharge/rehabilitation	Thrombolysis and thrombectomy were not indicated. The patient received antihypertensive therapy and dual antiplatelet therapy and was transferred to inpatient rehabilitation after 12 days with partial improvement.
Following months	Persistent cognitive and affective symptoms, including impaired concentration, recent-memory difficulties, marked mental fatigue, sleep disturbance, and depressive symptoms, with substantial occupational impairment.
5-year follow-up	The focal neurological deficit and post-stroke depression had largely improved, but memory difficulties, apathy/fatigue, and gait disturbance persisted.

Abbreviations: ChAdOx1 nCoV-19/AZD1222, COVID-19 Vaccine AstraZeneca; CT, computed tomography; CTA, computed tomography angiography; ECG, electrocardiography; MRI, magnetic resonance imaging; NIHSS, National Institutes of Health Stroke Scale; PF4, platelet factor 4; PCR, polymerase chain reaction; SARS-CoV-2, severe acute respiratory syndrome coronavirus 2; VITT, vaccine-induced immune thrombotic thrombocytopenia.

**Table 2:** Etiological evaluation and competing mechanisms in the presented case.

Domain Assessed	Findings in This Case	Interpretation
Clinical timing	Systemic symptoms within 24 h after vaccination; neurological onset on post-vaccination day 2.	Supports a temporal association and pharmacovigilance reporting, but does not establish causality.
Large-artery disease	CTA showed approximately 50% intradural left vertebral artery stenosis without large-vessel occlusion.	Potential artery-to-artery embolism or flow-related posterior circulation ischemia; not definitive as a sole etiology.
Small-vessel disease and vascular risk	History of hypertension, remote tobacco use, chronic lacunar infarcts, and mild leukoaraiosis.	Relevant vascular background findings do not establish a definitive alternative etiology.
Cardioembolism	ECG, inpatient cardiac assessment, prolonged rhythm monitoring, transthoracic echocardiography, and transesophageal echocardiography did not identify a definite cardioembolic source.	No definite cardioembolic mechanism was found.
VITT/TTS and venous thrombosis	Platelet counts remained within the reference range; D-dimer and anti-PF4 antibody testing were normal; dural venous sinuses were patent.	Classic VITT/TTS is unlikely because timing, platelet count, D-dimer/PF4 profile, and imaging were not typical.
SARS-CoV-2 infection and systemic inflammation	SARS-CoV-2 PCR was negative; routine biochemistry and inflammatory biomarker assessment did not reveal clinically significant abnormalities.	Acute SARS-CoV-2 infection or systemic inflammatory disease was not supported.
TOAST category	No single mechanism was proven after comprehensive evaluation; no definite alternative etiology was identified.	Best classified as ischemic stroke of undetermined etiology after comprehensive evaluation, while acknowledging the close temporal relationship with vaccination.

Abbreviations: CTA, computed tomography angiography; ECG, electrocardiography; PF4, platelet factor 4; PCR, polymerase chain reaction; SARS-CoV-2, severe acute respiratory syndrome coronavirus 2; TOAST, Trial of Org 10172 in Acute Stroke Treatment; TTS, thrombosis with thrombocytopenia syndrome; VITT, vaccine-induced immune thrombotic thrombocytopenia.

**Table 3:** Examples of acute arterial ischemic stroke occurring after COVID-19 vaccination in the literature and the present case.

Author/Year	Country	Age/Sex	Days After Vaccination	Vaccine/Context	Stroke/VITT	Other Thrombosis or Comments
Al-Mayhani/2021 [23]	England	35/F	6	ChAdOx1/AZD1222 post-vaccination stroke with VITT (first dose)	Arterial ischemic stroke/Yes	Venous thrombosis/Yes
Bayas/2021 [24]	Germany	55/F	7	ChAdOx1/AZD1222 post-vaccination stroke with VITT (first dose)	Arterial ischemic stroke/Yes	Venous thrombosis/Yes
Kenda/2021 [25]	Slovenia	51/F	7	ChAdOx1/AZD1222 post-vaccination stroke with VITT (first dose)	Arterial ischemic stroke/Yes	Venous thrombosis/No
Berlot/2022 [26]	Italy	69/F	2	ChAdOx1/AZD1222 post-vaccination stroke with VITT (first dose)	Arterial ischemic stroke/Yes	Venous thrombosis/No
Charidimou/2021 [27]	USA	37/F	10	Ad26.COV2.S vaccine	Arterial ischemic stroke/Yes	Venous thrombosis/Yes
Alammar/2021 [28]	Saudi Arabia	43/M	3	Post-vaccination stroke without VITT (first dose)	Arterial ischemic stroke/No	Venous thrombosis/No; severe hypertension
Assiri/2022 [29]	India	62/M	4	Post-vaccination stroke without VITT (first dose)	Arterial ischemic stroke/No	Venous thrombosis/No; severe hypertension
Corrêa/2021 [30]	Brazil	64/M	2	Post-vaccination stroke without VITT (first dose)	Arterial ischemic stroke/No	Venous thrombosis/No
Present case	Spain	63/M	2	Post-vaccination stroke without VITT (first dose)	Arterial ischemic stroke/No	Venous thrombosis/No; severe hypertension
Famularu/2021 [31]	Italy	87/F	1	BNT162b2	Arterial ischemic stroke/No	Second dose; atherosclerotic risk factors
Thomas/2023 [32]	Australia	30/M	1	BNT162b2	Arterial ischemic stroke/No	Third dose; atherosclerotic risk factors
Hidayat/2021 [33]	Indonesia	79/M	2	CoronaVac/Sinovac (first dose)	Arterial ischemic stroke/No	Atherosclerotic risk factors
Hidayat/2021 [33]	Indonesia	62/M	3	CoronaVac/Sinovac (first dose)	Arterial ischemic stroke/No	Atherosclerotic risk factors
Elaidouni/2022 [34]	Morocco	39/M	2	Sinopharm	Arterial ischemic stroke/No	Favorable clinical course

Abbreviations: Ad26.COV2.S, Johnson & Johnson/Janssen adenoviral-vector COVID-19 vaccine; BNT162b2, Pfizer-BioNTech COVID-19 vaccine; ChAdOx1/AZD1222, ChAdOx1 nCoV-19/AZD1222 vaccine; F, female; M, male; VITT, vaccine-induced immune thrombotic thrombocytopenia.

**Table 4:** SARS-CoV-2-related entities and stroke.

Entity/Syndrome	Prevalence/Estimate	Stroke Risk	Ischemic Stroke	CVST	Hemorrhage	Microcirculatory Disturbance
Acute SARS-CoV-2 infection (COVID-19)	1–5%	Increased	+	+	+	+
Post-COVID syndrome (PCS)	4.40 per 1000 vs. 3.25 per 1000 controls ^§^	Increased	+	+	+	+ ^†^
Neurologic PCS/Neuro-COVID *	Unknown	Not established	+	+	+	+ ^††^
PCS without neurologic syndrome	Variable ^‡^	Increased mainly after severe/hospitalized infection	+	+	+	+
Vaccination ^†††^	Variable ^§§^	Increased in rare cases	+	+	+	+

Abbreviations: COVID-19, coronavirus disease 2019; CVST, cerebral venous sinus thrombosis; PCS, post-COVID-19 syndrome; PCVS, post-COVID-19 vaccination syndrome; SARS-CoV-2, severe acute respiratory syndrome coronavirus 2. “+” indicates that the manifestation has been described. * Neurologic PCS/Neuro-COVID is used here to refer to neurological PCS presentations in which cognitive symptoms, brain vascular pathology, endothelial dysfunction, and microvascular effects have been proposed; reliable stroke-incidence data specifically within Neuro-COVID remain lacking. See refs. [71,90,196,215]. ^†^ See ref. [198]. ^‡^ Increased risk was mainly observed after severe or hospitalized SARS-CoV-2 infection; no increased long-term stroke risk was observed after community-managed infection in one national cohort study [191]. ^§^ See ref. [193]. ^§§^ Stroke reporting/estimates varied substantially across datasets: 12.2–65.3 reported stroke events per million administered doses in the EudraVigilance analysis [19] and 0.71 stroke cases per million administered doses in the Mexican nationwide descriptive study [21]. ^††^ Descriptive reports of neurologic PCS/Neuro-COVID include cerebrovascular and microvascular manifestations, but reliable prevalence and stroke-incidence estimates remain unavailable; see refs. [71,90,196,215]. ^†††^ The vaccination row summarizes reported events across vaccine platforms, including VITT/TTS-related and non-VITT events, and does not imply causality.

**Table 5:** SARS-CoV-2-related entities and stroke: evidence-graded pathophysiological mechanisms.

Mechanism/Evidence Level	Proposed Link to Stroke or Vascular Injury	Main Supporting References
Spike protein persistence or vaccine-induced epitope persistence (proposed/speculative for non-VITT arterial stroke)	May promote inflammation, endothelial dysfunction, effects on the blood–brain barrier, or immune response abnormalities in experimental or indirect studies. The clinical relevance of non-VITT arterial ischemic stroke after vaccination remains uncertain.	[61,68,69,70,71,151,152,159,160,161,162,163,164,165,166,174,199,200,218,219]
Inflammatory mediators of ischemic stroke (well supported in acute infection; indirect for vaccination)	Cytokine storm and inflammatory cytokine production may contribute to vascular injury and plaque instability, especially in acute or severe SARS-CoV-2 infection.	[60,147,153,154,155,166,220,221,222,223,224]
Coagulation dysfunction (well established in acute COVID-19 and VITT/TTS; variable for non-VITT stroke)	COVID-19 coagulopathy and VITT/TTS can promote thrombosis through different mechanisms. In the present case, normal platelet counts and normal D-dimer and anti-PF4 testing argue against classic VITT/TTS.	[13,14,15,16,23,24,25,26,27,40,43,44,45,47,56,108,111,118,132,137,142,151,156,157,171,183,219,225,226,227,228]
Endotheliopathy and microvascular injury (well supported in acute infection; proposed in PCS/PCVS and vaccination settings)	Endothelial activation may trigger coagulation pathways, reduce nitric oxide bioavailability, increase platelet adhesion, and impair microvascular function; extrapolating these effects to non-VITT post-vaccination stroke requires caution.	[69,109,117,120,138,146,147,152,163,166,167,168,169,170,171,172,173,174,175,196,198,215,229,230,231,232,233]
RAAS/ACE2 dysregulation and sympathetic activation (biologically plausible)	ACE2-related mechanisms may influence vascular tone, inflammation, procoagulant pathways, and blood pressure; in this case, severe hypertension may have been a trigger, a consequence, or a nonspecific stress response.	[120,234,235,236]
Immunological dysfunction and autoantibodies (established for VITT/TTS; proposed for other syndromes)	Anti-PF4 antibodies define VITT/TTS; other autoimmune or inflammatory mechanisms are reported but not proven in this patient.	[13,14,15,16,23,24,25,26,27,226,227,228]
Other mechanisms (hypothesis-generating)	BBB breakdown, mitochondrial dysfunction, microbiome dysbiosis, vasculitis, arterial stiffness, atherosclerosis, latent virus reactivation, cardiac dysfunction, hyperviscosity, capillary leak, and genetic traits have been proposed but remain unestablished explanations for this case.	[61,171,200,233,236,237,238,239]

Abbreviations: ACE2, angiotensin-converting enzyme 2; BBB, blood–brain barrier; PCS, post-COVID-19 syndrome; PCVS, post-COVID-19 vaccination syndrome; PF4, platelet factor 4; RAAS, renin–angiotensin–aldosterone system; SP, spike protein; TTS, thrombosis with thrombocytopenia syndrome; VEGF, vascular endothelial growth factor; VITT, vaccine-induced immune thrombotic thrombocytopenia.

## Data Availability

The data supporting the findings of this article are included within the manuscript.

## Supplementary Materials

The following supporting information can be downloaded at https://www.mdpi.com/article/10.3390/jcm15145487/s1, Supplementary File S1: CARE Checklist of Information to Include When Writing a Case Report.

## Abbreviations

Abbreviation	Definition
ACE2	angiotensin-converting enzyme 2
Ad26.COV2.S	Johnson & Johnson/Janssen adenoviral-vector COVID-19 vaccine
AEFI	adverse event following immunization
Ang II	angiotensin II
AT1R	angiotensin II type 1 receptor
BBB	blood–brain barrier
BNT162b2	Pfizer-BioNTech COVID-19 vaccine
ChAdOx1/AZD1222	ChAdOx1 nCoV-19/AZD1222 vaccine
COVID-19	coronavirus disease 2019
CT	computed tomography
CTA	computed tomography angiography
CVST	cerebral venous sinus thrombosis
ECG	electrocardiography
MRI	magnetic resonance imaging
NIHSS	National Institutes of Health Stroke Scale
NLRP3	NOD-, LRR- and pyrin domain-containing protein 3
PAI-1	plasminogen activator inhibitor-1
PCS	post-COVID-19 syndrome
PCVS	post-COVID-19 vaccination syndrome
PF4	platelet factor 4
PCR	polymerase chain reaction
RAAS	renin–angiotensin–aldosterone system
SARS-CoV-2	severe acute respiratory syndrome coronavirus 2
SASP	senescence-associated secretory phenotype
SP	spike protein
TF	tissue factor
TOAST	Trial of Org 10172 in Acute Stroke Treatment
tPA	tissue plasminogen activator
TTS	thrombosis with thrombocytopenia syndrome
VAERS	Vaccine Adverse Event Reporting System
VEGF	vascular endothelial growth factor
VITT	vaccine-induced immune thrombotic thrombocytopenia
vWF	von Willebrand factor
