# Supplementary material for: Posterior Circulation Ischemic Stroke Occurring After ChAdOx1 nCoV-19/AZD1222 Vaccination Without Evidence of Vaccine-Induced Immune Thrombotic Thrombocytopenia: A Case Report and Focused Narrative Review
Source: J Clin Med. 2026 Jul 13;15(14):5487. doi: 10.3390/jcm15145487 (PMC13412417; doi:10.3390/jcm15145487)
Supplement: Supplementary file 1 [file jcm-15-05487-s001.zip › jcm-4410766-supplementary.pdf]

Supplementary File S1. CARE Checklist of Information to Include When Writing a Case Report.

Manuscript title: Posterior Circulation Ischemic Stroke Occurring After ChAdOx1 nCoV-19/AZD1222 Vaccination Without Evidence of Vaccine-Induced Immune Thrombotic Thrombocytopenia: A Case Report and Focused Narrative Review

Checklist source: CARE Checklist of information to include when writing a case report (2013).

Topic	Item	CARE checklist requirement	Reported in final manuscript / comments
Title	1	The diagnosis or intervention of primary focus followed by the words "case report".	**Reported.** Title page, p. 1. The title includes "A Case Report and Focused Narrative Review".
Key Words	2	2 to 5 key words that identify diagnoses or interventions in this case report, including "case report".	**Partially reported.** Keywords, p. 2. The list identifies the diagnosis, vascular territory, vaccine, VITT, TOAST classification, and pharmacovigilance; however, it does not currently include the term "case report" and contains more than the 2–5 keywords recommended by CARE.
Abstract	3a	Introduction: what is unique about this case and what does it add to the scientific literature?	**Reported.** Abstract—Background, pp. 1–2.
Abstract	3b	Main symptoms and/or important clinical findings.	**Reported.** Abstract—Results, pp. 1–2; Section 3.1, Case Illustration, pp. 4–5.
Abstract	3c	The main diagnoses, therapeutic interventions, and outcomes.	**Partially reported.** The diagnosis and principal clinical, laboratory, and imaging findings are reported in the Abstract—Results, pp. 1–2. Therapeutic interventions and longitudinal outcomes are reported in Section 3.1 and Table 1, pp. 5–6, but are not summarized in the abstract.
Abstract	3d	Conclusion - what is the main take-away lesson(s) from this case?	**Reported.** Abstract—Conclusions, p. 2.
Introduction	4	One or two paragraphs summarizing why this case is unique (may include references).	**Reported.** Introduction, final paragraph, p. 3; Methods statement identifying the case report and focused narrative review, p. 3.
Patient Information	5a	De-identified patient-specific information.	**Reported.** Section 3.1, first paragraph, p. 4. Patient information is de-identified and unnecessary personal details are omitted.
Patient Information	5b	Primary concerns and symptoms of the patient.	**Reported.** Section 3.1, symptom-onset and neurological-examination paragraphs, p. 4; Table 1, pp. 5–6.
Patient Information	5c	Medical, family, and psycho-social history including relevant genetic information.	**Partially reported.** Section 3.1, first paragraph, p. 4, reports relevant medical and family history. Psychosocial and genetic history are not reported in the final manuscript.
Patient Information	5d	Relevant past interventions with outcomes.	**Not applicable / not reported.** No relevant previous therapeutic intervention and outcome are described before the index event. The absence of previous thrombosis, autoimmune disease, or recent heparin exposure is medical history rather than a prior intervention.
Clinical Findings	6	Describe significant physical examination and important clinical findings.	**Reported.** Section 3.1, neurological-examination paragraph, p. 4.
Timeline	7	Historical and current information from this episode of care organized as a timeline.	**Reported.** Table 1, pp. 5–6.
Diagnostic Assessment	8a	Diagnostic testing, such as physical examination, laboratory testing, imaging, surveys.	**Reported.** Section 3.1, pp. 4–5; Table 1, pp. 5–6; Section 3.2 and Table 2, pp. 6–7.
Diagnostic Assessment	8b	Diagnostic challenges, such as access to testing, financial, or cultural.	**Reported as applicable.** Sections 3.2–3.3 and Table 2, pp. 6–8, discuss etiological uncertainty, competing mechanisms, and the limitations of causal inference. No access, financial, or cultural barriers to testing were reported.
Diagnostic Assessment	8c	Diagnosis, including other diagnoses considered.	**Reported.** Section 3.2 and Table 2, pp. 6–7; Section 3.3, pp. 7–8. The final etiological classification is TOAST ischemic stroke of undetermined etiology after comprehensive evaluation; VITT/TTS and conventional competing mechanisms are considered.
Diagnostic Assessment	8d	Prognosis, such as staging in oncology, where applicable.	**Reported.** Section 3.1 follow-up paragraph and Table 1, pp. 5–6; Conclusions, p. 22.
Therapeutic Intervention	9a	Types of therapeutic intervention, such as pharmacologic, surgical, preventive, self-care.	**Reported.** Section 3.1, p. 5; Table 1, pp. 5–6. Antihypertensive therapy, dual antiplatelet therapy, and inpatient rehabilitation are reported.

Topic	Item	CARE checklist requirement	Reported in final manuscript / comments
Therapeutic Intervention	9b	Administration of therapeutic intervention, such as dosage, strength, duration.	**Partially reported.** Section 3.1 and Table 1, pp. 5–6, report the treatment categories and the 12-day neurology-ward course. Medication doses, strengths, and the duration of dual antiplatelet therapy are not specified in the final manuscript.
Therapeutic Intervention	9c	Changes in therapeutic intervention with rationale.	**Reported.** Section 3.1 and Table 1, pp. 5–6. Thrombolysis and thrombectomy were not indicated; medical treatment and rehabilitation were pursued.
Follow-up and Outcomes	10a	Clinician- and patient-assessed outcomes, if available.	**Reported.** Section 3.1 and Table 1, pp. 5–6. Partial improvement at transfer to rehabilitation and the 5-year clinical outcome are reported.
Follow-up and Outcomes	10b	Important follow-up diagnostic and other test results.	**Partially reported.** Section 3.1, Table 1, and Section 3.2, pp. 5–7, report the clinical follow-up and extended etiological evaluation. No specific follow-up neuroimaging or other longitudinal diagnostic test results are presented.
Follow-up and Outcomes	10c	Intervention adherence and tolerability - how was this assessed?	**Not specifically reported.** No adherence problem or treatment intolerance is described in the available clinical documentation.
Follow-up and Outcomes	10d	Adverse and unanticipated events.	**Reported as absent.** No treatment-related adverse or unanticipated event is reported. Persistent cognitive, affective, fatigue, and gait symptoms are presented as post-stroke outcomes/sequelae rather than adverse effects of treatment.
Discussion	11a	A scientific discussion of the strengths and limitations associated with this case report.	**Reported.** Strengths and limitations are addressed across Sections 3.2–3.4, pp. 6–12, and the Conclusions, p. 22.
Discussion	11b	Discussion of the relevant medical literature with references.	**Reported.** Sections 3.4 and 4.1–4.4, pp. 10–21; References.
Discussion	11c	The scientific rationale for any conclusions, including assessment of possible causes.	**Reported.** Sections 3.2–3.4, pp. 6–12; Section 4.4, pp. 17–21; Conclusions, p. 22.
Discussion	11d	The primary take-away lessons of this case report in a one-paragraph conclusion.	**Reported.** Conclusions, p. 22.
Patient Perspective	12	The patient should share their perspective in one to two paragraphs on the treatment(s) they received.	**Not reported.** A formal patient-perspective statement was not collected for this retrospective, anonymized case report.
Informed Consent	13	Did the patient give informed consent? Please provide if requested.	**Reported.** Back matter—Informed Consent Statement, p. 22. Written informed consent for publication of the clinical details and neuroimaging findings was obtained from the patient.

Note: Because the article combines a case report with a focused narrative review, some CARE items are reported across the case presentation, etiological assessment, and discussion sections. Items that are only partially reported or not applicable are explicitly identified.
